# Ligand Recognition by the TPR Domain of the Import Factor Toc64 from *Arabidopsis thaliana*

**DOI:** 10.1371/journal.pone.0083461

**Published:** 2013-12-31

**Authors:** Rashmi Panigrahi, Abdussalam Adina-Zada, James Whelan, Alice Vrielink

**Affiliations:** 1 School of Chemistry and Biochemistry, University of Western Australia, Crawley, Western Australia, Australia; 2 ARC Centre of Excellence in Plant Energy Biology, University of Western Australia, Crawley, Western Australia, Australia; 3 Department of Botany, School of Life Science, La Trobe University, Bundoora, Victoria, Australia; Russian Academy of Sciences, Institute for Biological Instrumentation, Russian Federation

## Abstract

The specific targeting of protein to organelles is achieved by targeting signals being recognised by their cognate receptors. Cytosolic chaperones, bound to precursor proteins, are recognized by specific receptors of the import machinery enabling transport into the specific organelle. The aim of this study was to gain greater insight into the mode of recognition of the C-termini of Hsp70 and Hsp90 chaperones by the Tetratricopeptide Repeat (TPR) domain of the chloroplast import receptor Toc64 from *Arabidopsis thaliana* (*At*). The monomeric TPR domain binds with 1∶1 stoichiometry in similar micromolar affinity to both Hsp70 and Hsp90 as determined by isothermal titration calorimetry (ITC). Mutations of the terminal EEVD motif caused a profound decrease in affinity. Additionally, this study considered the contributions of residues upstream as alanine scanning experiments of these residues showed reduced binding affinity. Molecular dynamics simulations of the TPR domain helices upon peptide binding predicted that two helices within the TPR domain move backwards, exposing the cradle surface for interaction with the peptide. Our findings from ITC and molecular dynamics studies suggest that *At*Toc64_TPR does not discriminate between C-termini peptides of Hsp70 and Hsp90.

## Introduction

Non-globular proteins, which contain repeated structural motifs arranged in tandem, are ubiquitous in nature. Many of these proteins have extended structures with exposed interaction surfaces. The common scaffold is known to interact with a variety of ligands [1] and the binding of the ligand does not induce any structural rearrangement [1], [2]. Armadillo repeats (ARM), Ankyrins (Ank), Leucine-rich repeats (LRR), HEAT repeats and Tetratricopeptide repeats (TPR) are some examples of repeat protein [1], [3], [4]. Structural and binding studies of the Hsp70 and Hsp90 organizing protein (Hop) [5], [6] have provided some knowledge of the interactions between the TPR domain and peptides, however, the role of conformational changes in these interactions has not been well characterized.

In this study, we focus on Tetratricopeptide Repeat (TPR) repeats and their versatility with respect to ligand recognition. These structural domains act as interaction scaffolds and mediators of multi-protein complexes and are found in all kingdoms of life [7]. TPR repeats form the 20 most common folds in the Pfam database [8]. They consist of multiple repeats of degenerate 34 amino acids, forming the canonical helix-turn-helix fold. Typically proteins with this motif contain 3–16 sequential TPR motifs, arranged in a tandem array. The anti-parallel packing of the helices forms a grooved surface with concave and convex faces, with ligand binding usually occurring within the concave surface [9]. TPR interaction with their ligand is usually specific. This specificity is provided by the unique geometry of the binding pocket and ionic, hydrogen bond and hydrophobic interactions between amino acids residues of the TPR and the ligand. TPR domains span a range of oligomeric states from monomers [6], [10] to higher oligomers [11]–[13]. Nuclear protein ssn6 [14], chromatin associated protein CDC23 [15] and mitotic chromosome disjunction protein nuc2 + [16] were among the first to be identified as TPR containing proteins. Furthermore, proteins with TPR domains play an essential role in the import of proteins into mitochondria [17], chloroplast [18] and peroxisomes [19].

Proteins destined to the chloroplast are nuclear encoded, synthesized in the cytosol, and transported as precursor proteins (or preproteins), usually with NH_2_-terminal targeting sequence called transit peptides [20]. As both the mitochondria and chloroplast co-exist in plant cells, the sorting of protein between these organelles is a unique aspect of plant cell biology, and thus place a higher degree of stringency of protein targeting and sorting compared to non-plant cells. In chloroplasts, the process of recognition and translocation is initiated by subunits of the multimeric protein import complex called translocon at the outer envelope of chloroplast (Toc). This Toc core is comprised of the channel type, Toc75 [21], membrane anchored GTPases Toc159 [22], Toc34 [23] and an integral membrane protein Toc64 [24]. Topology analyses of Toc64 suggest that it consists of an amidase domain and a C-terminal TPR domain. The protein spans the membrane three times and positions the TPR domain facing the cytosol [20], [25]. The TPR domain of Toc64 is composed of three TPR repeats followed by a solvation or capping helix (Figure S1A) [26]. Association of Toc64 with preproteins was found to be chaperone mediated [25], [27]. Toc64 from *Pisum sativum* was found to interact with the C-terminal peptides of both Hsp70 and Hsp90 from wheat germ lysate, however, a preference was observed for interaction with the C-terminus of Hsp90 from human [25]. In the above interaction studies, Toc64, Toc64_TPR, and the C-terminal peptide of Hsp90 were matrix immobilized. It was also observed from *in vivo* experiments that the TPR domain of Toc64 exhibited a stronger interaction with Hsp90 whereas the transmembrane region acts as substrate for Hsp70 [25]. Thus, the TPR domain of Toc64 acts as a docking site, preferentially for Hsp90 bound preproteins, whereas Hsp70 was thought to be non-essential for interaction with preproteins [25], [27]. The EEVD motif in the C-terminus of Hsp70 and Hsp90 families is highly conserved in all eukaryotes. Although this motif anchors to the TPR domain by a dicarboxylate clamp mechanism, residues N-terminal to this sequence have also been reported to contribute towards the specificity of interaction through hydrophobic and van der Waals contacts as in case of Hop [6]. The interaction analysis of the EEVD peptide with TPR domain showed a drastic reduction in binding affinity in Hop on removal of the above N terminal residues [5].

In the present study, we characterized the interaction of the TPR domain from *At*Toc64 with C-terminal peptides from Hsp70 and Hsp90 by using biophysical approaches as a continuation of studies carried out by Qbadou S, *et al.* [28]. Using isothermal titration calorimetry studies and molecular dynamics simulations, we have investigated the mode of interaction of *At*Toc64 with C-terminal octapeptides of Hsp70 and Hsp90. *At*Toc64_TPR interacts with the above octapeptides with similar affinity and 1∶1 stoichiometry. Noticeably, point mutations of any of the last five residues of the peptides to alanine cause significant abolishment in peptide binding. Using molecular dynamics, we have delineated the contribution of residues involved in interactions and the dynamics mediating binding events. These studies showed that the Hsp70/90 octapeptide bound conformations are mediated by the outward movement of the terminal helices of the TPR, exposing the inner surface of the cradle for interaction with the peptide.

## Materials and Methods

### Expression clone of the TPR domain of *At*Toc64

**Figure 1:**
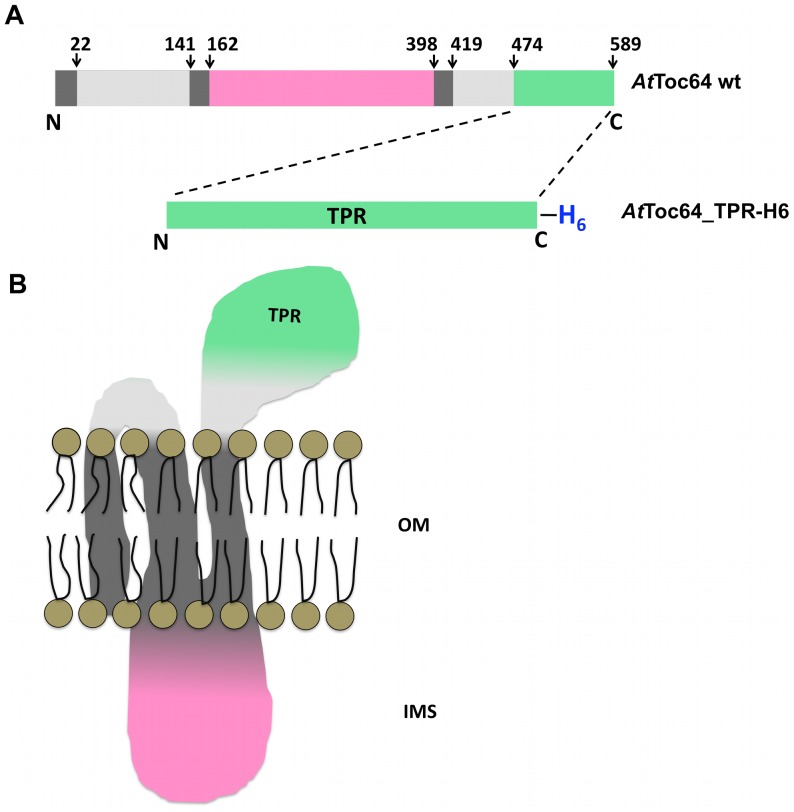
Topological model of Toc64 from *Arabidopsis thaliana*. **A.** Domain organization in *At*Toc64 from the uniprot database (http://www.uniprot.org/uniprot/Q9LVH5#section_name). Different regions shown are helical transmembrane regions (dark grey), cytoplasmic exposed regions shown (light grey), regions exposed to the intermembrane space (pink) and TPR domain (green). The TPR domain of the protein expressed for this study is shown in green with a hexahistidine tag at the C-terminus. **B.** Topological model of the domain organization. OM: outer membrane, IMS: Intermembrane space.

An expression clone for the TPR domain of *At*Toc64 was constructed using a cDNA encoding the full-length protein (accession number NP_188424, At3g17970). The region encompassing the TPR domain of *At*Toc64 was composed of residues 474–589 (Figure 1). PCR amplification of this region used the forward primer (5′- TTA**CCATGG**CCGAGATTGCCAAAGAGAAGGGTAA -3′, *Nco1* site underlined), and the reverse primer (5′- TTCA**CTCGAG**CTGGAATTTTCTCAGTCTCTCTGC -3′, *Xho1* site underlined). The PCR amplified product was digested with *Nco1* and *Xho1* and inserted into the pETM10 expression vector (Invitrogen), digested with the same restriction enzymes to generate the *At*Toc64_TPR_pETM10 expression plasmid encoding the TPR domain containing a C-terminal hexa-histidine tag (*At*Toc64_TPR-H6).

### Expression in *E. coli*

Overexpression of the TPR sequence was performed in *E. coli* BL21(DE3) (Novagen). 1 µg of plasmid DNA was used to transform 50 µL of competent cells. Single colonies were grown in Luria broth medium at 37°C supplemented with 40 µg/µL of kanamycin (Amresco). Protein expression was induced by the addition of 1 mM isopropyl- β-D-thiogalactopyranoside (IPTG). After 12 hours of induction at 20°C, cells were harvested by centrifugation, and the pellet obtained was used for protein purification.

### Protein purification

Recombinant expressed TPR domain was purified using nickel affinity chromatography (Ni-NTA). Briefly, 12 g of pellet was resuspended with 400 mL of lysis buffer (50 mM Hepes, 500 mM KCl, 10% glycerol, pH 7.3). The resuspended pellet was lysed using an Emulsiflex C5 high-pressure homogenizer (Avestin) at 13000 p.s.i. The lysate was clarified by centrifuging at 22,700× g for 45 min followed by filtering the supernatant through a 0.22 µm membrane. The supernatant was loaded onto a 5 mL HisTrap FFcrude column using an ÄKTA purifier FPLC system (GE Healthcare). The Ni-NTA bound TPR was eluted using a concentration gradient of 40 mM to 1 M of imidazole (pH 7.3) and the concentration of the protein was quantified by measuring the absorbance at 280 nm using a calculated extinction coefficient of 1.09 M^−1^ cm^−1^. The protein purity was evaluated using standard SDS-PAGE analysis. The purified protein was concentrated using an Amicon centrifugal filter unit (30 kDa cutoff).

### Relative molecular mass estimation by size exclusion chromatography

A superdex 75 prep grade size exclusion chromatography column (GE Healthcare) was equilibrated with superdex buffer (25 mM Hepes, 100 mM KCl, pH 7.3). The column was calibrated using gel filtration standards (Alcohol dehydrogenase, *M_r_* 150,000: void volume; Bovine serum albumin, *M_r_* 66,000 (1); Ovalbumin *M_r_* 44,000 (2), Carbonic anhydrase *M_r_* 29,000 (3); Lysozyme *M_r_* 14,000 (4). 2 mL of concentrated protein at 8 mg/mL was applied to the equilibrated column and eluted using the above superdex buffer. The relative molecular masses of the peaks obtained were calculated using a logarithmic interpolation. The elution peak corresponding to non-aggregated *At*Toc64_TPR-H6 was pooled and concentrated in the buffer systems required for various biophysical experiments.

### Peptide synthesis

Synthetic C-terminal octapeptides to Hsp70 (GPTIEEVD) and Hsp90 (TSRMEEVD) were designed. In addition, shortened versions of these peptides were designed containing only the C-terminal pentapeptide sequences: Hsp70_C5 (IEEVD), Hsp90_C5 (MEEVD). Additionally, alanine mutations of each of the amino acids in the octapeptides were designed representing a library of 16 Ala-substituted peptides. Two random sequences of five residues (ASDTM) and eight residues (DMTSRGTQ) were also designed for negative control experiments. All the above peptides were synthesized and provided at >85% purity by Biomatik (Canada).

### Circular Dichroism studies (CD)

Far-UV CD spectra corresponding to peptide bond absorption were recorded from 185 to 260 nm at 10°C using a Jasco-810 spectropolarimeter. Spectra were collected for 10 µM of protein in 10 mM of sodium phosphate buffer pH 7.3 in a Quartz SUPRASIL cuvette (Hellma) with a path length of 1 mm. Measurements were made with an increment step of 0.5 nm, an integration time of 4 sec per step and a bandwidth of 2 nm. The signal due to buffer alone was subtracted from that of the protein.

The proportions of secondary structures of the protein was estimated from the [Θ] values between 190 and 240 nm using the DichroWeb server [29] (http://dichroweb.cryst.bbk.ac.uk/html/home.shtml) and the CDSSTR algorithm [30]–[32]

The helical content of *At*Toc64_TPR-H6 was calculated from the molar ellipticity at 208 and 222 nm [33] using the following equation:
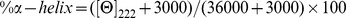
The change in molar ellipticity was monitored at 222 nm while varying the temperature from 10–80°C at a rate of 1°C/min. The thermally denatured sample was cooled to 10°C at the same rate to observe the effect of temperature on the folding of recombinant expressed *At*Toc64_TPR-H6.

### Dynamic Light Scattering studies (DLS)

DLS experiments were carried out with a Zetasizer Nano ZS (Malvern). 35 µM of protein in the superdex buffer was used for this study. Measurements were performed in triplicate consisting of 10 acquisitions per run.

### Analytical Ultra-centrifugation (AUC)

These experiments were conducted on a Beckman XL-A analytical centrifuge, which was equipped with An60-Ti rotor and absorbance optics [34]. The experiments were carried out using 70 µM of protein samples in superdex buffer (390 µL) with superdex buffer (400 µL) as reference separately loaded into a double sector centrepiece and built up in the rotor. Protein samples were monitored continuously by UV absorbance at 280 nm. Prior to the start of centrifugation, the rotor was equilibrated to 20°C and the vacuum was brought below 10 micron. The rotor was then set to spin at 60,000 rpm for 16 hrs. A total of 300 scans were collected. The acquired data were analysed using the SEDFIT program [35]. The c(s) method implemented in the program was used for the data analysis, where c(s) is the sedimentation coefficient distribution function of the macromolecule. The physical parameters of the sample solution used for the data analysis were partial specific volume (0.74 ml/g), buffer density (1.005 g/ml) and viscosity (0.01002 P). This provides excellent resolution and sensitivity for characterizing sample homogeneity. A confidence level of p = 0.95 was used while solving the size distributions.

### Isothermal Titration Calorimetry (ITC)

Peptides were dissolved in the superdex buffer at 6–14 mM concentration and 302 µL were titrated against 1.4 ml of protein at 0.1–0.3 mM in a VP-ITC Microcal Instrument (GE Healthcare). Titrations were carried out at 20°C using 30 injections of 10 µL each injected at interval of 200 seconds. Injections were continued beyond saturation levels to allow determination of the heats of ligand dilution. The non-linear least square curve-fitting algorithm (Microcal Origin) was used for data fitting. After subtraction of the heat of dilution, three floating variables: stoichiometry (N), binding constant (K_d_) and change in enthalpy of interaction (ΔH) were obtained. For subsequent alanine scan experiments the stoichiometry was fixed at N = 1.

### Molecular Dynamics Simulation Studies

#### Initial Geometry

A reliable model of the TPR domain for *in silico* studies was obtained by submitting the *At*Toc64_TPR protein sequence for automated protein structure modeling using the I-TASSER pipeline [36], [37]. A model was constructed using multiple threading alignments, which avoided bias towards a particular structural model as in homology modeling [38]. The molecular systems for protein-ligand complexes were built using the high-resolution crystal structure of 1ELW, the TPR domain of Hop in complex with the C-terminal octapeptide of Hsp70 [6] (obtained from the Protein Data Bank) as templates and a model obtained from the I-TASSER server. The TPR domains from Hop and *At*Toc64 exhibited 50% sequence identify. The starting structures were refined in 200 independent FlexPepDock simulations, which consisted of a low-resolution pre-optimization step followed by a high-resolution refinement and high-resolution mode simulations using Rosetta FlexPepDock [39], [40]. Thus two near native models of protein-peptide complexes were constructed and used for molecular dynamics simulations. In total, three molecular systems were prepared: *At*Toc64_TPR receptor (Apo), *At*Toc64_TPR receptor with C-term Hsp70 (octapeptide) bound (T_C70) and *At*Toc64_TPR receptor with C-term Hsp90 (octapeptide) bound (T_C90). Ramachandran plots (RAMPAGE, http://mordred.bioc.cam.ac.uk/~rapper/rampage.php) [41], of the modeled structures are found in the supplementary materials .

#### Preparation for simulation

Graphics Processing Unit (GPU) accelerated Assisted Model Building with Energy Refinement (AMBER) suite version 12 [42] associated with the latest all-atom ff12SB force field was used for simulation studies [43] . The starting structures were neutralized using Na^+^ and Cl^−^ ions and hydrogen atoms positioned using the *tleap* module from AMBERTOOLS12. The protein was centered in a solvent truncated octahedron box, which was made of TIP3P (3-point charged) triangulated water molecules with a 12 Å cut off in all directions [44]. The total number of atoms including water molecules was approximately 20,000 across various systems. The systems were minimized using a two-phase energy minimization approach which included 2500 cycles of steepest descent and 2500 cycles of conjugate gradient with solute atoms restrained by harmonic potentials with force constants of 50 kcal mol^−1^ Å^2^. This was followed by 5000 steps of unrestrained whole system minimization. 50 ps of density equilibration with weak harmonic restraints of 2 kcal mol^−1^ Å^2^ on the solute molecule was performed followed by unrestrained equilibration for 500 ps under constant pressure and temperature conditions. All simulations were performed using the SHAKE algorithm [45] with constraints on hydrogen-linked bonds (allowing a tolerance of 0.0001). To evaluate long-range electrostatic interactions, the Particle Mesh Ewald (PME) method [46] was used with a cut-off of 9 Å. An integration time step of 2 fs was used to numerically solve Newton's equations of motion. Langevin dynamics was used to maintain a constant temperature of 300 K throughout the simulations. All the simulations were performed using the PMEMD module in AMBER12. Production runs were carried out for 50 ns in an explicit solvent environment and with an isothermal-isobaric (NPT) ensemble.

#### Analysis of trajectory

After the production runs were completed, each of the trajectories were analyzed based on variation in kinetic and potential energies using the *ptraj* program. Root-mean-square deviation (rmsd) and atomic positional fluctuation per residue (rmsf) were analyzed to understand the overall conformational changes throughout the trajectory. The number of hydrogen and other non-bonded bond interactions were calculated using HBPLUS [47]. Interactions of arginine or lysine side chains with oxygen atoms of aspartate or glutamate residues which were from 3–4 Å in distance and exhibited angles (donor – H – acceptor) less than 90° were not classified as hydrogen bonds, but considered as electrostatic interactions. In addition, the solvent accessible surface area and binding energies for the protein-peptide interactions were calculated using NACCESS [48] and MM/PB(GB)SA tools [49], [50] respectively. Computational Alanine scanning was performed with the peptide, in order to understand the contribution of individual amino acid residues towards binding. When calculating the difference in free energies (ΔΔG) between the wild type and the mutants, the results of using Generalized Born calculations (GB) were taken into consideration, as they are well suited for protein-ligand and protein-protein interaction calculations.

These values were calculated according to the following equation:
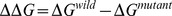
For all of the above analyses, the last 5 ns of the trajectory was analyzed. The APBS software was used to compute the electrostatic potential surface [51]. Ligplot was used to map the hydrogen and hydrophobic bonding patterns between the peptide residues and the residues of TPR involved in interaction [52]. Visual Molecular Dynamics [53] was used for visualizing the trajectories of the simulations of the three systems. The dictionary of protein secondary structure (DSSP) [54] was used to assign the secondary structure per amino acid along different trajectories.

Principal Component Analysis (PCA) was also performed in order to substantiate the above results [55]. GROMOS 4. 5 [56] was used on the protein backbone atoms (Cα) to obtain the collective coordinates for protein motions from covariance matrices in the form of sets of eigen values (amount of motion) and eigen vectors (direction of motion). In this way all the linearly correlated motions were analyzed.

Correlated atomic motion in the apo and ligand bound forms were obtained by analyzing the dynamical cross correlation map (DCCM) of Cα atoms using Bio3D [57], [58]. This provided a means to understand the correlation of motions of neighbouring and/or distant residues [59]

All graphical representations were generated using PyMOL [60].

## Results

### Purification and characterization of TPR domain of *At*Toc64

**Figure 2:**
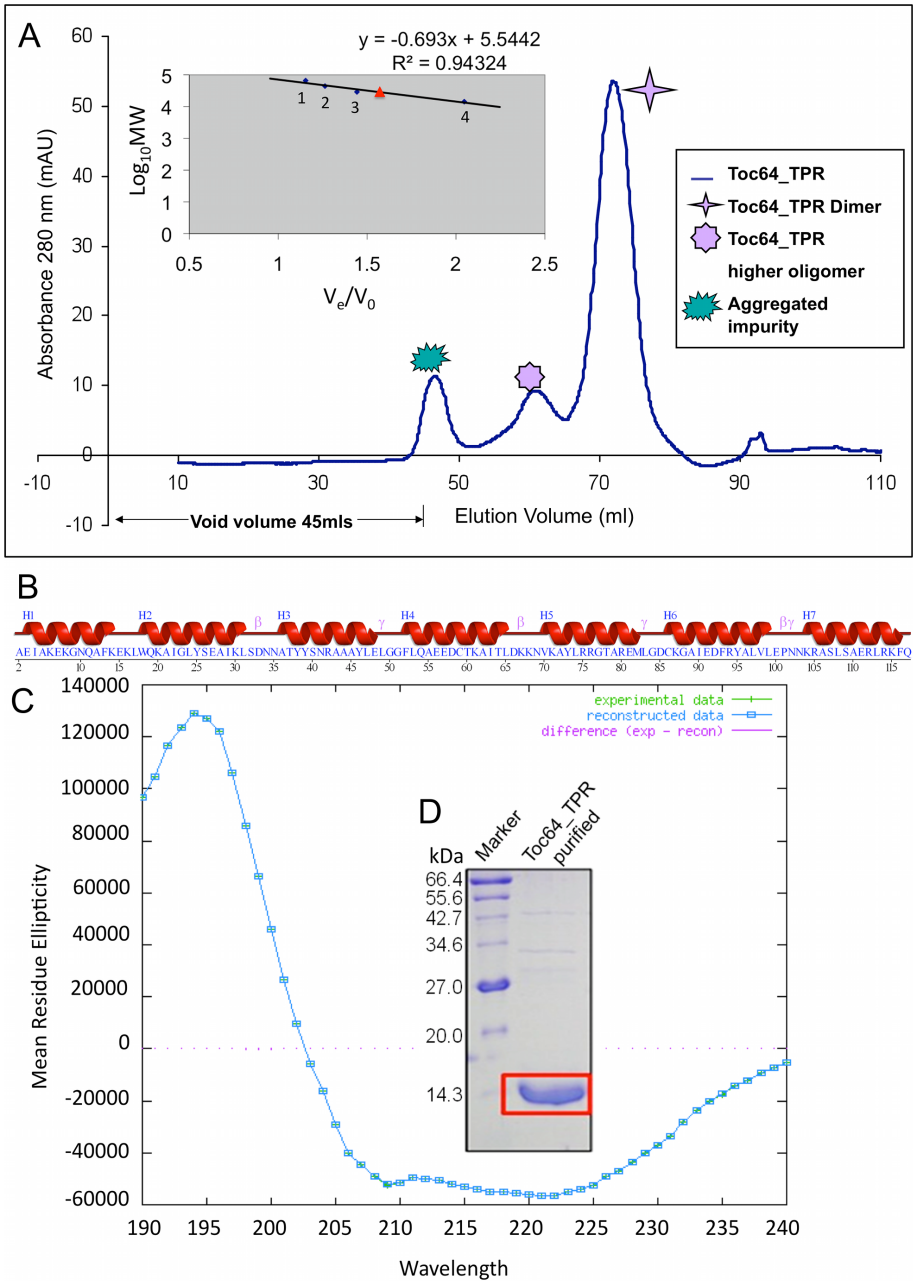
Biophysical characterization of *At*Toc64_TPR-H6 using size exclusion chromatography and circular dichroism. **A.** Elution profile of *At*Toc64_TPR-H6 from size exclusion chromatography using a Superdex 75 prep grade column. The different shaped stars represent different forms of the protein as described in the figure. The Ve/Vo versus LogMW plot for superdex standards is shown as an inset. Numbers 1–4 represent different standard proteins used as described in the methods. Red triangle represents *At*Toc64_TPR-H6. **B.** Schematic representation of secondary structure of *At*Toc64_TPR. **C.** CD spectrum profile of the protein obtained after analysis with DICHROWEB.

Recombinant *At*Toc64_TPR-H6 (Figure 1) was cloned, over-expressed and purified using Ni-NTA affinity chromatography. The purity was accessed by SDS-PAGE where a single band, corresponding to 14.3 kDa, was observed (Figure S1B). The observed molecular mass agreed with that predicted from the sequence of his-tagged *At*Toc64_TPR. Further size exclusion chromatography was performed which showed that the protein exists predominantly as a dimer calculated from gel filtration protein standards (Figure 2A). The dimer fractions were pooled, concentrated and used for further studies. Based on these results, we hypothesize that the TPR domain is either globular and dimeric or elongated and monomeric.

The TPR repeats are known to adopt an alpha-helical solenoid structure; this is supported by analysis of the protein sequence by PSIPRED [61], [62] (Figure 2B). Circular dichroism spectra can be used to estimate the regular secondary structural elements in a protein. The spectral signature revealed that protein adopted a predominantly (80%) helical structure (Figure 2C), in agreement with that observed in the PSIPRED prediction. Thermal denaturation experiments showed that the protein had a T_m_ of ∼35°C (Figure S1C).

### Estimation of polydispersity by DLS and AUC

In order to understand the oligomeric state of the protein, DLS studies were performed. This analysis on a 35 µM solution of freshly purified recombinant *At*Toc64_TPR-H6 showed that the protein was predominantly monodispersed with a polydispersity value of 13%. Storage of the protein at 4°C for 24 hours caused an increase in the extent of polydispersity (52%) as compared to the freshly purified sample. AUC experiments were carried out to independently establish the homogeneity of protein molecules in solution and determine their molecular mass [63]. The protein existed as a mixture of monomeric and dimeric forms, where the former was predominantly present (Table 1). These results also explained the observed sample polydispersity by DLS; this polydispersity was irreversible. Additionally, AUC experiments were performed in combination with the two peptides (octapeptides). Interestingly, it was observed that in all cases the monomeric form was predominant (Table 1). The result of the AUC studies suggests that the behaviour of *At*Toc64_TPR-H6 by size exclusion chromatography is likely due to the non-globular, ellipsoid shape and not due to the presence of dimers in solution. The higher oligomers observed in the AUC is likely due to nonspecific self-association of protein molecules with time.

**Table 1:** Analytical ultracentrifugation.

Components	Sedimentation Coefficient	Molecular mass (kDa)	Monomer %
*At*Toc64_TPR	1.60±0.17	15±2	67
*At*Toc64_TPR with C-terminal Hsp70 octapeptide	1.9±0.3	**	83
*At*Toc64_TPR with C-terminal Hsp90 octapeptide	1.9±0.2	14.9±2	71

Analytical ultracentrifugation experiments were performed at 20°C in superdex buffer. As the frictional coefficient was high (f/f_0_ = 1.54) in the Hsp70 bound form hence adequate determination of the molecular mass was not possible. However the sedimentation coefficient obtained suggests that it is a monomer.

### ITC characterization of peptide interaction with *At*Toc64_TPR-H6

**Figure 3:**
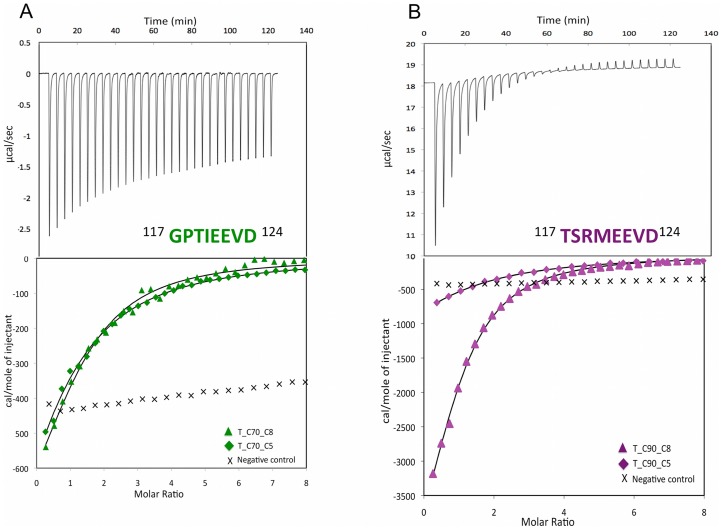
Binding isotherms for the interaction of *At*Toc64_TPR-H6 with Hsp70 and Hsp90. The ITC isotherms obtained for the Hsp70 octapeptide binding to *At*Toc64_TPR-H6 (in green) and the Hsp90 octapeptide binding to *At*Toc64_TPR-H6 (in magenta). The bottom panels show the curves obtained for titration of the octapeptides of Hsp70/90 into TPR (triangles); the C-terminal pentapeptides of Hsp70/90 into TPR (diamonds) and random peptides into TPR (crosses).

In order to understand the energetics of binding between the C-termini of Hsp70/Hsp90 and the TPR domain of *At*Toc64, binding studies using ITC were performed [64], [65]. The curves obtained were fit using a 1∶1 binding model. The binding stoichiometries (n) were found to be 1.2 and 0.94, when protein at 300 µM in the superdex buffer was titrated with 10 mM Hsp70 octapeptide or 14 mM Hsp90 octapeptide solutions respectively at 20°C. Similar experiments were carried with pentapeptide versions of Hsp70 and Hsp90, wherein n values were 1.2 and 1.04 respectively. Hence in all of the above cases, the thermodynamics of binding events exhibited exothermic behaviour. The heat of reaction per injection was calculated from the area under the peak, which gradually decreased with complex formation and reached the heat of dilution of the respective peptide when the protein was saturated. After ∼20 and ∼11 injections in case of Hsp70 and Hsp90 respectively, no unbound TPR was present. The thermodynamic parameters and the final titration curve (Figure 3A and 3B) were computed, resulting in similar binding affinities for the two octapeptides to TPR. Reducing the length of the peptide to the C-terminal five residues containing the EEVD motif decreased the binding affinity two and half fold in the case of Hsp70 and four fold in the case of Hsp90 (Table 2). The Gibb's free energy (ΔG) of the above binding events were similar in magnitude to each other, ranging from −4.90 to −4.94 kcal/mol. In the case of the octapeptide of Hsp90, a favourable enthalpic contribution to binding was observed (ΔH = −6.18 kcal/mol) with a decrease in entropy (ΔS = −4.33 cal/mol•K). This is characteristic of an enthalpy driven process for binding of the peptide to the protein, where possible changes in conformation of one or both components in the binding event is expected. In the case of the octapeptide of Hsp70, the enthalpic contribution to the binding event was quite low (ΔH = −1.0 kcal/mol) accompanied by an increase in entropy (ΔS = 13.2 cal/mol•K). An increase in entropy was also seen in the binding of the pentapeptide of both Hsp70 (ΔS = 9.29 cal/mol•K) and Hsp90 (ΔS = 5.03 cal/mol•K), suggesting an entropy driven event which may be due to a combination of the release of water molecules from the binding site upon ligand binding and a classical hydrophobic effect. In summary, a favourable enthalpic binding process, in spite of a decrease in entropy, have aided the binding of the protein with the Hsp90 octapeptide; whereas a favourable entropic process, despite a decrease in enthalpy have aided the binding of protein with Hsp70 octapeptide.

**Table 2:** Thermodynamic parameters obtained for TPR-Hsp interaction using isothermal titration calorimetry.

Systems	N	ΔG (kcal/mol)	ΔH (kcal/mol)	TΔS (kcal/mol)	K_d_ (µM)
T_C70_C5	0.93+/−0.13	−4.37	−1.63	2.72	568
T_C70_C8	1.2+/−0.12	−4.9	−1.0	3.86	230
T_C90_C5	1.04+/−0.13	−4.17	−2.68	1.47	800
T_C90_C8	0.94+/−0.04	−4.94	−6.18	−1.26	218

N is the stoichiometry; ΔG is the calculated change in Gibb's free energy; ΔH is the change in enthalpy; ΔS in the change in entropy and K_d_ is the binding affinity.

### Experimental alanine scanning

**Figure 4:**
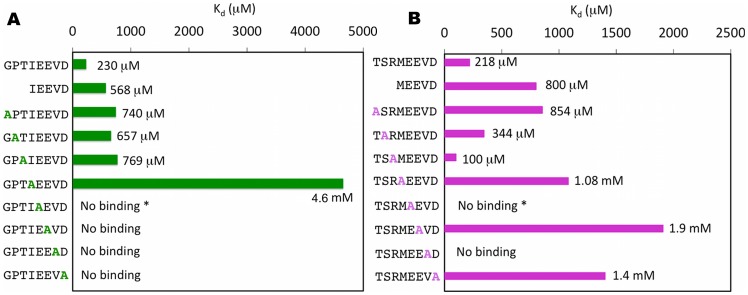
The ITC binding data from alanine scanning mutagenesis of the peptide interaction with *At*Toc64_TPR-H6. **A.** Data using the C-terminal octapeptide from Hsp70. **B.** Data using the C-terminal octapeptide of Hsp90. Asterik (*) suggests that the heat change during binding event is quite low and the signal to noise ratio is high. Hence the K_d_ is considered as no binding.

The EEVD motifs in the peptides have been shown previously to act as an anchor to the protein [5]. As both the octapeptides bound to the protein with similar affinity, it was of interest to investigate the contribution of each residue towards the interaction. For this work alanine scanning mutagenesis was carried out on each of the octapeptides used for the study and the binding was characterized by ITC studies (Figure 4). For this description, the N-terminal Gly residue is termed Gly^117^, and the following residues are arranged in ascending order, *e.g.* Pro^118^, Thr^119^, Ile^120^, Glu^121^, Glu^122^, Val^123^, Asp^124^, in case of Hsp70 and a similar nomenclature for Hsp90 is used. Alanine substitution of Asp^124^ abolished binding in Hsp70 and caused a significant decrease in case of Hsp90, suggesting the occurrence of electrostatic interactions. A strong contribution of hydrophobic interactions to the binding was hypothesized because of a significant increase in K_d_ or the complete loss of binding in the case of Ile^120^/Met^120^ and Val^123^ respectively. As expected, mutation of Glu^121^ and Glu^122^ to Alanine abolished or significantly reduced the binding affinity for Hsp70 and Hsp90 respectively. Mutation of the first four N-terminal residues in both the peptides to Alanine caused a decrease in binding affinity with the exception of the Arg^119^ in case of Hsp90 (Figure 4, Table S1).

### Simulation studies

**Figure 5:**
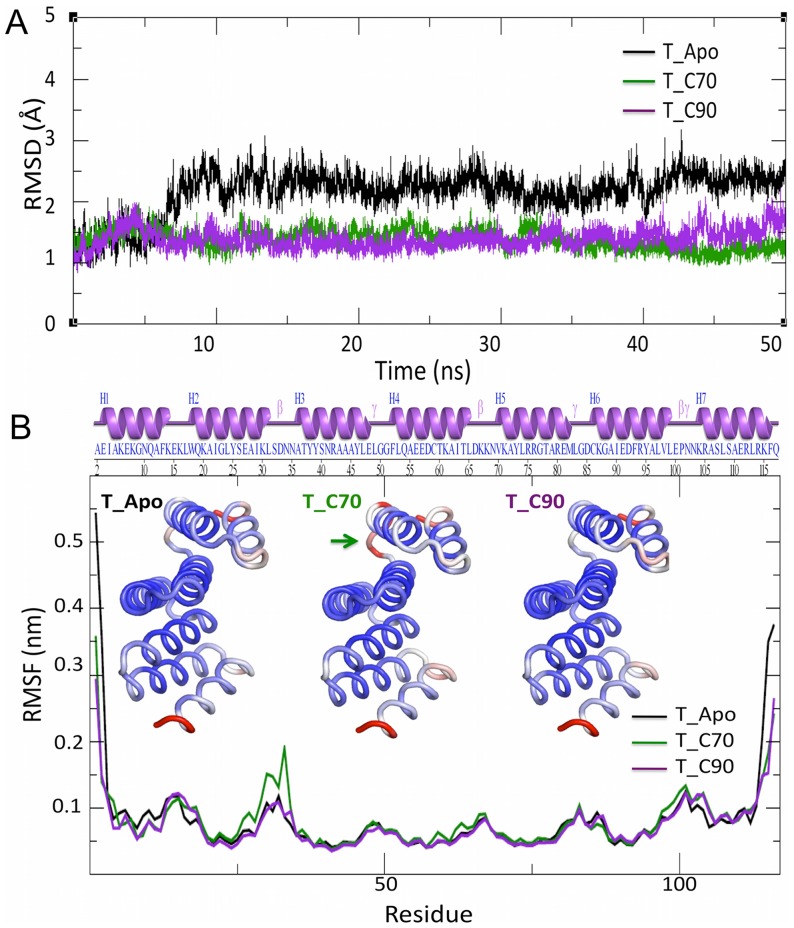
Analysis of the molecular dynamics trajectory obtained after 50 A. Root mean square deviation (RMSD) plot for the apo TPR receptor (black trace), the Hsp70 C-terminal octapeptide bound form of the receptor (T_70: green trace) and the Hsp90 C-terminal octapeptide bound form of the receptor (T_C90: magenta trace). **B.** The atomic positional fluctuation (RMSF) plot obtained for each of the above systems. The loop with high B factors in T_C70 is shown with a green arrow.

The quality of the models generated were checked using Ramachandran plot where ∼94% of the residues were present in the favoured region (Figure S3, S4 and S5), Further the RMSD between the *At*Toc64_TPR modelled using ITASSER and the TPR domain of Hop (1ELW) was found to be 1.4 Å (Figure S2). Three systems were simulated based on the isothermal and isobaric (NPT) ensemble for 50 ns each: apo *At*Toc64_TPR (apo), *At*Toc64_TPR with the octapeptide of Hsp70 bound (T_C70) and *At*Toc64_TPR with the octapeptide of Hsp90 bound (T_C90). The RMS fluctuation for each system at the end of the equilibration was minimal. The overall structural stability of the systems throughout the simulation was assessed by calculating the RMSD of the Cα atoms from the appropriate starting structures for each simulation. The average Cα-RMSD were found to be 2.15 Å, 1.38 Å, and 1.39 Å for the apo, T_C70 and T_C90 systems respectively (Figure 5A). This suggested that the complexed structures were stable and retained their overall structure during the simulation. Further analysis of the three independent simulations for each of the systems indicated that, in all cases, the cradle topology of the TPR was maintained. An analysis of Cαatomic positional fluctuation (RMSF) for each of the systems showed that the N and C termini exhibited higher temperature factors (B factor). Additionally, higher B factors were also observed for TPR residues 29–33 in the T_C70 system, corresponding to a loop connecting the H2 and H3 helices (Figure 5B). Finally, the RMSF of the terminal residues were decreased relatively upon ligand binding.

### Analysis of protein-peptide interactions

**Figure 6:**
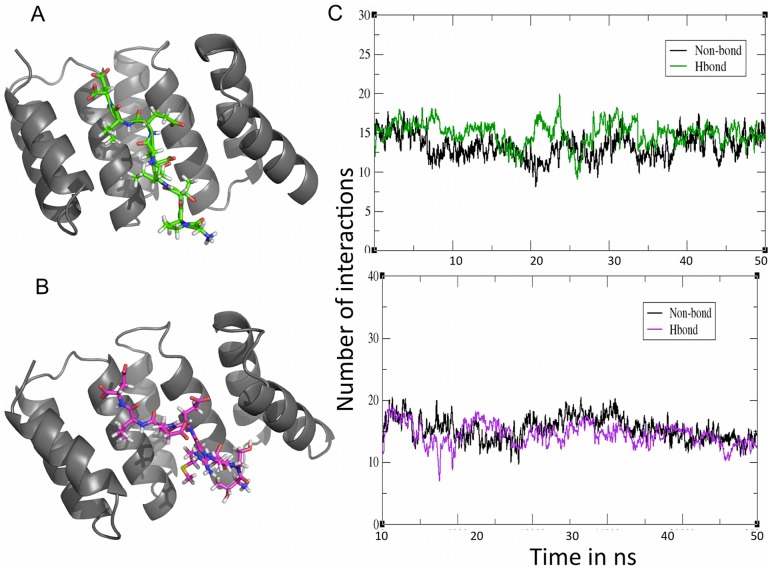
Characterization of the average structures obtained after simulation of the complexes. **A.** The TPR domain with bound octapeptide from Hsp70 (green) **B.** The TPR domain with bound octapeptide from Hsp90 (magenta) **C.** Number of interactions in the T_C70 system (green) and in the T_C90 system (magenta) during the simulations.

The concave surface of the TPR cradle was crucial for peptide binding as observed in the average structures (Figure 6A and 6B). This is exemplified through intermolecular hydrogen bonding and nonbonding interactions with the residues that line the inner surface of the cradle. Approximately 15 interactions of each type are found in both of the peptide bound forms (Figure 6C). Notably, two types of hydrogen bonding interactions existed on the protein-peptide interface: sequence specific interactions, which involved the side chains of the peptide residues and sequence independent interactions, which involved the peptide main chain. Intra-residue interactions are also observed and are likely to be necessary to maintain the proper conformation of the peptides necessary for interactions with the protein.

#### Carboxylate clamp

**Figure 7:**
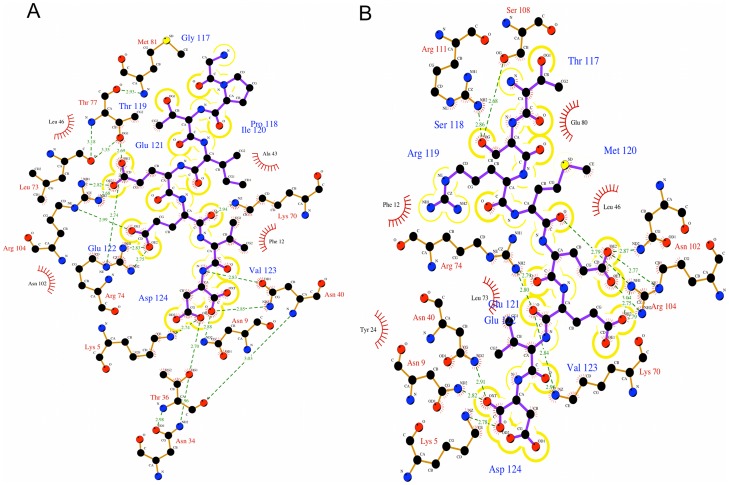
Interactions occurring in the protein-peptide interface generated by Ligplot. **A.** Interactions between the TPR domain and the C-terminal octapeptide of Hsp70; **B.** Interactions between the TPR domain and C-terminal octapeptide of Hsp90. The peptide is shown in purple bonds and the protein in brown bonds.

**Figure 8:**
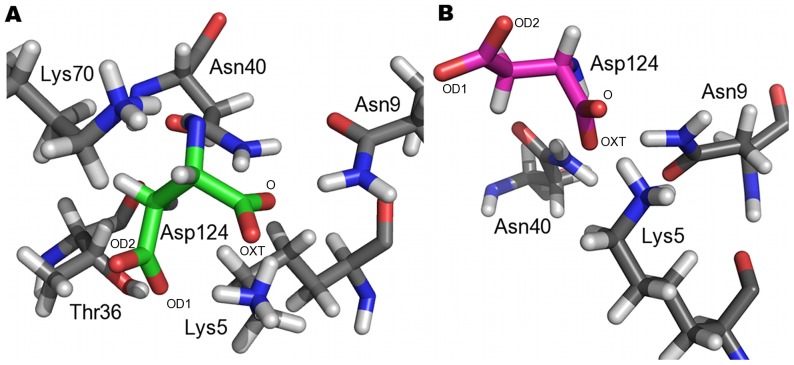
The carboxylate clamp. **A.** Key interacting residues of the TPR domain with the carboxylate of Asp^124^ of Hsp70 (shown in green bonds); **B.** Key interacting residues of the TPR domain with the carboxylate of Asp^124^ of Hsp90 (shown in magenta bonds). The oxygen atoms of the carboxylate moieties are labeled. The TPR domain is shown with grey bonds.

Interaction analysis of the crystal structure of TPR domains from Hop liganded with the C-terminal octapeptide and pentapeptide of Hsp70 and Hsp90 respectively, have shown that the carboxylate moieties of the highly conserved terminal Asp residue of the peptides form electrostatic interactions with the protein residue [6]. This has been referred to as the two-carboxylate clamp. The conserved Asp^124^ in both the complexes are clamped to the TPR by the terminal carboxylate moieties and held in place by a myriad of interactions to the protein (Figure 7A, 7B). In case of the T_C70 system, the two carboxylates are held in place by hydrogen bonding interaction with Lys^5^ and Asn^9^ of helix H1. Additionally, the main chain carboxylate is stabilized by interaction with Asn^40^ of helix H3 whereas Thr^36^ of helix H3 and Lys^70^ of helix H5 help in stabilizing the side chain carboxylate (Figure 8A). In the T_C90 system, the terminal carboxylate of Asp^124^ are clamped by hydrogen bonding interactions with Lys^5^, Asn^9^ of helix H1 and Asn^40^ of helix H3 (Figure 8B). Thus the carboxylate clamp, which is highly conserved in both the systems, anchors the peptides to the TPR.

#### Other key hydrogen bonding interactions

The hydrogen bonding interactions from the TPR domain to the peptide in each complex are directed predominantly towards side chains, hence exploit sequence-specific features. Residues N terminal to Glu^121^ of the C-term Hsp70 peptide do not display any hydrogen bonding interaction with the TPR. In T_C90 system, Ser^118^ forms hydrogen-bonding interaction with Ser^108^ and Arg^111^and Met^120^ displays hydrogen bonding interaction with Arg^104^. Side chain hydrogen bonding interactions of Glu^121^ exist with side chains of Arg^74/^/Arg^74^, Thr^77^/Asn^102^ and Arg^104^/Arg^104^) in the T_C70/T_C90 systems respectively (Figure 7A, 7B). The carboxylate oxygen atoms of Glu122 form hydrogen bond acceptors for Lys^70^, Arg^74^ and Arg^104^ nitrogen containing groups in both the systems and additionally with Arg^119^ in the T_C90 system. Val^123^ acts as hydrogen bond acceptor for Lys^70^, exclusively in T_C90 system.

#### A groove for Valine

Among the charged residues of the EEVD motif in the peptides, valine is the only hydrophobic residue. The Val^123^ is held in place by a number of van der Waals and hydrophobic interaction with different residues of the TPR domain, which form a groove. The interacting residues forming van der Waals contacts are Asn^9^ and Asn^40^ in case of T_C70 system and Asn^40^, Lys^70^ and Arg^74^ in case of T_C90 system. Similarly, Phe^12^ and Ala^43^ in case of T_C70 and Phe^12^ in case of T_C90 form hydrophobic interactions with Val^123^.

#### Intrapeptide interactions

**Figure 9:**
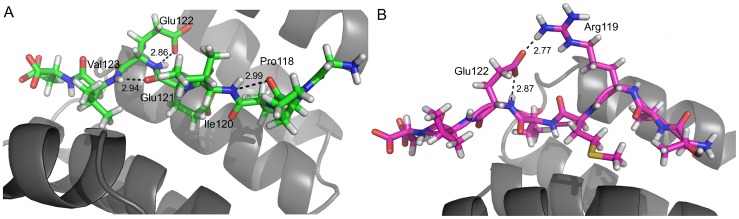
Key intrapeptide interactions. **A.** Intrapeptide interaction in the T_C70 system (coloured green) **B.** Intrapeptide interaction in the T_C90 system (coloured magenta). The TPR domain is shown as a ribbon diagram in grey.

Intrapeptide interactions play a key role in giving a particular conformation to the peptide. This helps in the proper presentation of the peptide residues, which aid in interaction to the protein (Figure 9A, 9B). In the T_C70 system, the key intrapeptide hydrogen bonding interactions are as below: the main chain nitrogen of Ile^120^ with main chain oxygen of Pro^118^; the main chain nitrogen of Val^123^ with main chain oxygen of Glu^121^ and the main chain nitrogen of Glu^122^ with side chain oxygen of Glu^122^. Similarly in the T_C90 system, the key intrapeptide hydrogen bonding interactions are: the amino group of Arg^119^ with the side chain oxygen of Glu^122^ and the main chain nitrogen of Glu^122^ with its own side chain oxygen.

### Computational alanine scanning

Though absolute free energies from simulations are not achievable [66], the underlying trend caused due to mutation has been reliably captured. Computational Alanine scanning was performed on the trajectories of the two complex systems. Six of the eight residues in the each of the peptides were mutated individually to Alanine. The terminal amino acids could not be mutated by MMPB/GBSA due to limitation of the program. The calculated binding free energies for the Hsp70 and Hsp90 liganded systems were found to be −12.6 and −13.8 kcal/mol respectively. Mutations of Val^l23^, Glu^122^, Glu^121^, Met120 and Ile^120^ individually to Ala caused a decrease in binding, which was in accordance with our experimental ITC findings (Table 3). Alanine mutation of Arg^119^ slightly improved binding as observed in our experiments. In contrast, mutation of Pro^118^ and Ser^118^ to Alanine had only a minimal effect on binding, a result that differed from the ITC results. The slight change in conformation of the peptides caused by the mutations may have resulted in changes to the binding affinity; these have not been taken into consideration by the computational alanine scanning studies. However, the differences found in the later case was quite small, and hence the trend of change in binding free energies could be considered acceptable. In all computational studies the secondary structure of the protein is conserved all throughout the simulation and there is very little change between alpha, pi and 3_10_ helices (Figure S5).

**Table 3:** Computational Alanine Scanning for both ligand bound systems.

**Hsp70 mutants**	ΔΔG
G**A**TIEEVD	−0.39
GP**A**IEEVD	−0.04
GPT**A**EEVD	−2.01
GPTI**A**EVD	−17.6
GPTIE**A**VD	−15.9
GPTIEE**A**D	−3.66
**Hsp90 Mutants**	
T**A**RMEEVD	−0.14
TS**A**MEEVD	−1.21
TSR**A**EEVD	−3.08
TSRM**A**EVD	−12.0
TSRME**A**VD	−3.37
TSRMEE**A**D	−3.97

Point mutations to alanine were performed computationally for each residue (in bold) of the Hsp70 and Hsp90 octapeptides. Changes in binding free energy (ΔΔG) in kcal/mol is calculated using MMGBSA.

### Dynamic cross-correlation map (DCCM)

Dynamic cross-correlation maps suggest that distinct correlation exist between the helices (Figure S7). In the ligand unbound form, the majority of residues show a positive correlation between H1 and H2; H3 and H4; H4 and H5 (Figure S7A). Fewer residues show positive correlation between H5 and H6. Negative correlated motion is observed in H1 with respect to H4 and H6. Also the segment, residues between 40–60, which comprises of a part of H3, a loop and a part of H4 show negative correlation with H7. Similarly, H5 shows negative correlation with H7. Ligand binding reduces all the observed inter helical positive correlations, further enhancing negative correlated motions (Figure S7B, S7C). In T_C70, additional negative correlation is seen between H6 and the region consisting of residues 60–70 (Figure S7B).

### Principal component analysis (Essential dynamics)

**Figure 10:**
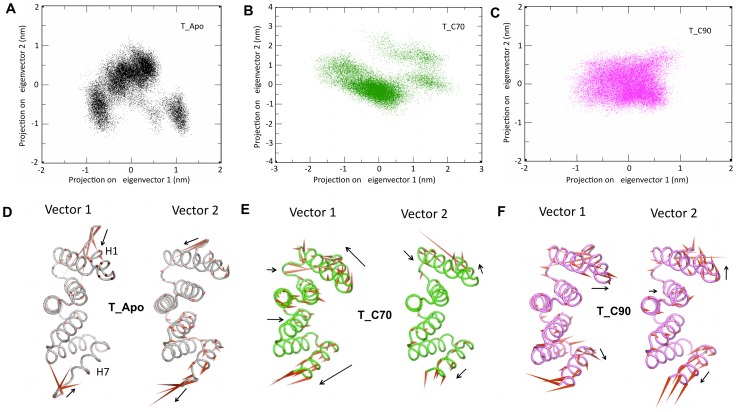
Principal component analysis for the three systems. Essential dynamics -2D projection of individual trajectories with their first two eigen vectors: vector 1 and vector 2 (**A–C**) and their corresponding porcupine plots (**D–F**) for T_Apo (black), T_C70 (green) and T_C90 (magenta). Porcupine plots of the three systems displayed with a cone model. The length and orientation of the cone (red) is positively correlated with the magnitude and direction of motion.

Although DCCM, is a good way to analyze motions between the pair of atoms, the complexity of collective principal atomic motion could not be visualized. To understand this, essential dynamics was performed. The overall protein motions were decomposed into a set of eight eigenvectors. The first two eigenvectors (most significant principal components, PC1 and PC2) accounted for ∼65% of the total fluctuation. The 3D plot of these two components projected, with the potential energy on the z-axis, is represented as a 2D plot (Figure 10A–C). The plots depict that there is clearly more conformational space sampled in the case of Apo form. The dominant motions in the apo form are confined to the terminal residues of the H1 and H7 helices (Figure 10D), which correlate with the rmsd and positional fluctuation plots. The distributions of both the complexes are smaller than that observed in the apo form. In the T_C90 system, the conformational spread is more restricted (Figure 10C) than observed for the T_C70 system (Figure 10B). An analysis of the individual principal components in the complexes suggests that protein in the peptide bound forms do not show loss of alpha helicity and suggests that peptide binding may act to stabilize the TPR. The most prominent motions are observed in the H1 and H7 helices with very little motion in concave inner surface of the TPR cradle (Figure 10D–F).

## Discussion

In the present study, we purified recombinant *At*Toc64_TPR-H6 to homogeneity. Analytical ultracentrifugation experiments estimate that the TPR domain exists predominantly as a monomer. Isothermal calorimetry studies indicate that the domain interacts with the C-terminal regions of Hsp70 and Hsp90 with similar micromolar affinity and 1∶1 stoichiometry. Molecular dynamics studies have been performed to provide atomic level descriptions for the protein–peptide interactions. The terminal aspartate in both Hsp70 and Hsp90, is anchored to the TPR by a dicarboxylate clamp, supporting previous findings [6]. Electrostatic potential surface representations of the ligand bound form of *At*Toc-TPR shows that the peptides are bound to a predominantly positively charged surface within the cradle of the TPR domain. In the case of Hsp70, the N-terminal residues are exposed and not interacting with the protein (Figure S8). Alanine scanning mutations of residues 120 to 124 in both peptides significantly perturb binding to the TPR domain. Intramolecular hydrogen bonding interactions between different residues of the peptides are observed, which might be necessary for providing suitable conformations to the peptides needed for binding to the receptor. Though hydrogen bonds, electrostatic and hydrophobic contacts exist, van der Waals interactions play a major role in positioning the EEVD motif. Alanine scanning mutation of either Gly^117^ or Pro^118^ using ITC reduces the binding affinity of the peptide, however the MD studies do not show that these residues interact specifically with the TPR domain. A possible explanation for this observation may be that residues contribute to the ideal conformation of the peptide necessary for interaction with the protein or they can prevent sampling of unwanted conformational space by the peptide. Proline commonly adopts a cis conformation. Glycine, as a small and achiral amino acid, can occupy a larger volume of conformational space without unfavourable steric interactions with other amino acids. Because of these unique features, glycine and proline residues often occur in turns and loops [67], [68].

Using MMPB/GBSA for computational alanine scanning, the change in binding energy due to mutation of Gly117 and Pro118 to alanine could not be captured. The plausible reason for the above observation is that in MD experiments, the peptide is initially docked to the protein. The binding energy change upon mutation can be reliably captured for residues of the peptide, which directly interact with the protein. Although residues such as glycine and proline in the Hsp70 C-terminal peptide, do not interact directly with the TPR domain, they are likely to play an important role in providing the required conformation to the peptide for recognition by the TPR domain. Thus the binding energy changes (ΔΔG) cannot be reliably computed for alanine mutations of residues, which are not directly interacting with the protein.

Unfavorable entropic contributions upon ligand binding are often compensated for by increased dynamic motion in distant regions of the protein. Essential dynamics shows motion in the terminal helices upon binding with either peptide. The H1 and H7 helices move backwards in order to expose the inner surface of the cradle for peptide binding. It is known that TPRs have a rigid conformation [26]. In contrast, this study provides evidence that the change in curvature by the concerted movement of secondary structural elements may be necessary for ligand binding. This observation supports a recent study on a TPR containing protein, MamA, where 3 Å radial movement by two N-terminal TPR motifs was observed in different crystal structures upon ligand imitator binding [12]. Unlike the case of Hop, where there are two TPR domains with specificity for Hsp binding, proteins such as CHIP and CNS1 [28], [69] recognize both Hsp70 and Hsp90 with single TPR domain. In CHIP, I^120^ (Hsp70)/M^120^ (Hsp90) are accommodated in a hydrophobic pocket, which is absent in Hop [6]. In our case, Ile^120^ and Val^123^ sandwich the Phe^12^ of *At*Toc_TPR, thus enhancing the interaction through van der Waals contacts.

In the current work, the two peptides used for this study exhibit a conserved EEVD motif, however residues N-terminal to this conserved sequence differ between Hsp70 (sequence: G^117^P^118^T^119^I^120^) and Hsp90 (sequence: T^117^S^118^R^119^M^120^). Despite these differences, the two peptides bind to the *At*Toc64_TPR with similar affinity. The differences in the observed binding mechanism (entropy *vs* enthalpy driven reactions) is likely to be a consequence of the contributions of the different residues located at the N-terminus, where peptides with different sequences bind with similar affinity to the same protein. Interestingly, the pentapeptide versions of Hsp70 (sequence: I^120^E^121^E^122^V^123^D^124^) and Hsp90 (sequence: M^120^E^121^E^122^V^123^D^124^) demonstrate entropically driven interactions as observed by ITC studies, although with weak micromolar affinity than the octapeptides. Additionally the residues glycine and proline do not interact with the protein in the case of the TPR-Hsp70 system. It is tempting to conclude from the above observed results that the interactions involved with the N-terminal residues of the Hsp90 octapeptide, namely threonine, serine and arginine, contribute towards an enthalpically driven interaction. Additionally, one might hypothesize that the interaction of hydrophobic residues such as I^120^ and M^120^ might assist in the release of water molecules from the interaction interface.

Furthermore, computational studies using essential dynamics suggests that a higher conformational spread is observed in the TPR-Hsp70 system than in the TPR-Hsp90 system resulting in an entropy driven process in the former system. The observed flexibility in the terminal helices, H1 and H7, upon ligand binding may further contribute to the entropy of the system. However, in the TPR-His90 system, the interaction with the N-terminal residues (T^117^S^118^R^119^) appears to dominate over the entropy due to the increased flexibility thus resulting in an enthalpy driven process.

This is the first study reported till date, where we throw light on the AtToc64_TPR and C-term Hsp70/Hsp90 interaction at a molecular level using *in vitro* studies with ITC and *in silico* studies with molecular dynamics. Our *in vitro* and *in silico* studies suggest that *At*Toc64_TPR can recognize the C-terminal octapeptide of both Hsp70 and Hsp90 with similar affinity and mutation of residues within this peptide to alanine destabilizes the interactions.

## Supporting Information

Table S1 **Alanine scanning for both ligands (Hsp70/90) using ITC.**
(DOCX)

Figure S1 **Characterization of the** ***At*** **Toc64_TPR-H6.** **A.** Cradle structure of the 3-TPR domain. Helices are numbered as H1 to H7. Each TPR forms a helix turn helix structure which repeats itself three times followed by a solvation or capping helix. **B.** 16% denaturing SDS PAGE gel showing the purification of *At*Toc64_TPR-H6 after Ni-NTA chromatography. The band observed in the red box indicates the presence of *At*Toc64_TPR-H6. **C.** Thermal denaturation curve of *At*Toc64_TPR-H6. T_m_ was calculated to be ∼35°C.
(TIF)

Figure S2 **Overlay of the ITASSER modelled structure on the Hop crystal structure.** Tube depiction of the superposition of the TPR model obtained from ITASSER on the high-resolution crystal structure of the TPR domain from Hop complexes with the Hsp70 octapeptide (1ELW). The modelled structure of the TPR domain is shown in blue color and that of the crystal structure is shown in yellow. The RMSD was found to be 1.36 Å, suggesting that the modeled structure was quite reliable for use in MD studies.
(TIF)

Figure S3 **Ramachandran plot of modelled** ***At*** **Toc64_TPR (Apo).**
(TIF)

Figure S4 **Ramachandran plot of C-Hsp70 (octapeptide) bound form (T_C70).**
(TIF)

Figure S5 **Ramachandran plot of C-Hsp90 (octapeptide) bound form (T_C90).**
(TIF)

Figure S6 **Secondary structure map of all the trajectories created by DSSP.** Color codes: green represents alpha helices, black represents turns, grey represents 3_10_ helices and white represents coils.
(TIF)

Figure S7 **Dynamic cross-correlation maps.** **A.** Apo, **B.** C-Hsp70 bound form of TPR and **C.** C-Hsp90 bound for of TPR using Cα atoms.
(TIF)

Figure S8 **Molecular surface representations.** **A.** C-Hsp70 octapeptide is bound to *At*Toc64_TPR; **B.** C-Hsp90 octapeptide is bound to *At*Toc64_TPR. The averaged pdb of the final 5ns of the simulation from each trajectory was used to create these maps. Electronegative and electropositive charges are colored in red and blue respectively.
(TIF)
