# Supplementary material for: Ligand Recognition by the TPR Domain of the Import Factor Toc64 from Arabidopsis thaliana
Source: PLoS One. 2013 Dec 31;8(12):e83461. doi: 10.1371/journal.pone.0083461 (PMC3877065; doi:10.1371/journal.pone.0083461)
Supplement: Table S1 — Alanine scanning for both ligands (Hsp70/90) using ITC. (DOCX) [file pone.0083461.s001.docx]

**Table S1:**

Alanine scanning for both ligands (Hsp70/90) using ITC.

| Hsp70 mutants | N | ∆G (kcal/mol) | ∆H (kcal/mol) | T∆S (kcal/mol) | K_d_ (µM) |
| --- | --- | --- | --- | --- | --- |
| APTIEEVD | 1 | -4.2 | -1.0 | 3.2 | 740 |
| GATIEEVD | 1 | -4.2 | -2.4 | 1.8 | 657 |
| GPAIEEVD | 1 | -4.1 | -1.6 | 2.5 | 769 |
| GPTAEEVD | 1 | -3.1 | -5.5 | -2.4 | 4651 |
| GPTIAEVD | - | - | - | - | No binding |
| GPTIEAVD | - | - | - | - | No binding |
| GPTIEEAD | - | - | - | - | No binding |
| GPTIEEVA | - | - | - | - | No binding |
| Hsp90 mutants |  |  |  |  |  |
| ASRMEEVD | 1 | 7.5 | 8.8 | 1.3 | 854 |
| TARMEEVD | 1 | -1.3 | 1.7 | 3.0 | 344 |
| TSAMEEVD | 1 | -5.4 | -2.2 | 3.2 | 100 |
| TSRAEEVD | 1 | -4.0 | -2.3 | 1.7 | 1080 |
| TSRMAEVD | - | - | - | - | No binding* |
| TSRMEAVD | 1 | -3.7 | -2.3 | 1.4 | 1908 |
| TSRMEEAD | - | - | - | - | No binding* |
| TSRMEEVA | 1 | -3.8 | -0.4 | 3.4 | 1402 |

Synthetic octapeptides were used, where point mutation of individual residue into alanine, helped in understanding the contribution of the same towards the thermodynamics of binding. Asterik (*) suggests that the heat change during binding event is quite low and the signal to noise ratio is high. Hence the K_d_ was considered as no binding.
