# Supplementary figures and images for: Ligand Recognition by the TPR Domain of the Import Factor Toc64 from Arabidopsis thaliana
Source: PLoS One. 2013 Dec 31;8(12):e83461. doi: 10.1371/journal.pone.0083461 (PMC3877065; doi:10.1371/journal.pone.0083461)

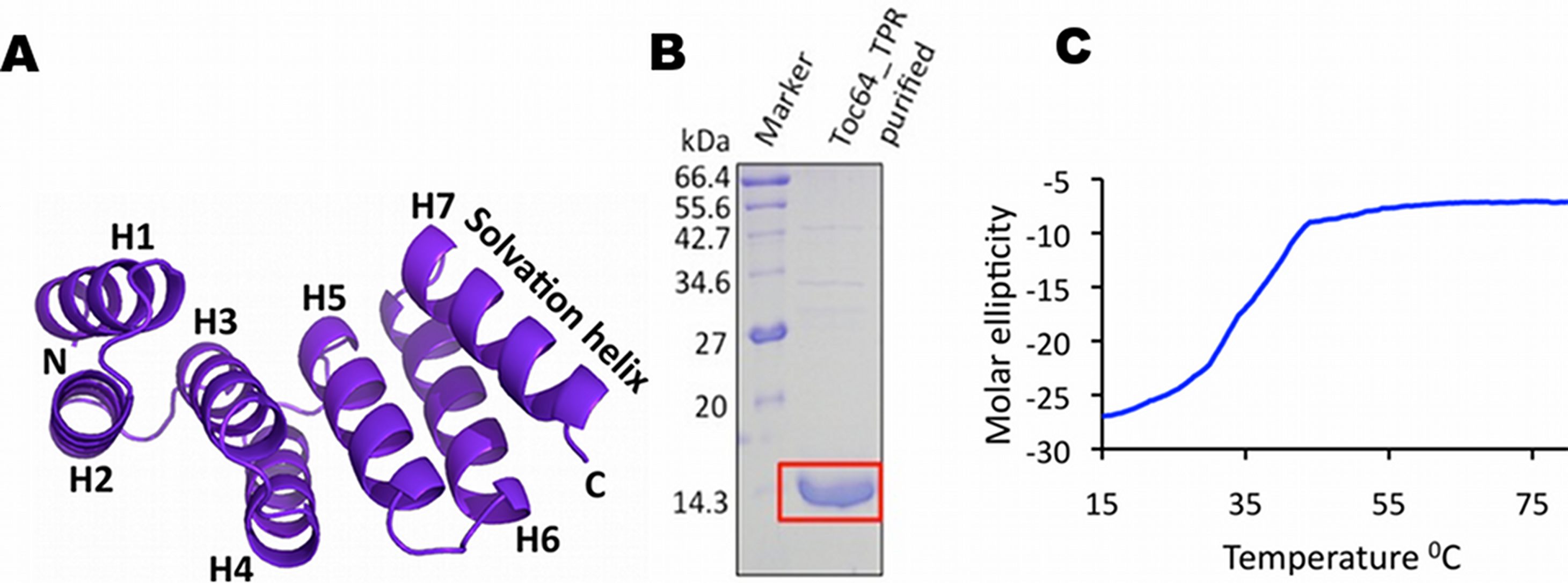

Supplement: Figure S1 — Characterization of the At Toc64_TPR-H6. A. Cradle structure of the 3-TPR domain. Helices are numbered as H1 to H7. Each TPR forms a helix turn helix structure which repeats itself three times followed by a solvation or capping helix. B. 16% denaturing SDS PAGE gel showing the purification of AtToc64_TPR-H6 after Ni-NTA chromatography. The band observed in the red box indicates the presence of AtToc64_TPR-H6. C. Thermal denaturation curve of AtToc64_TPR-H6. Tm was calculated to be ∼35°C. (TIF) [file pone.0083461.s002.tif]

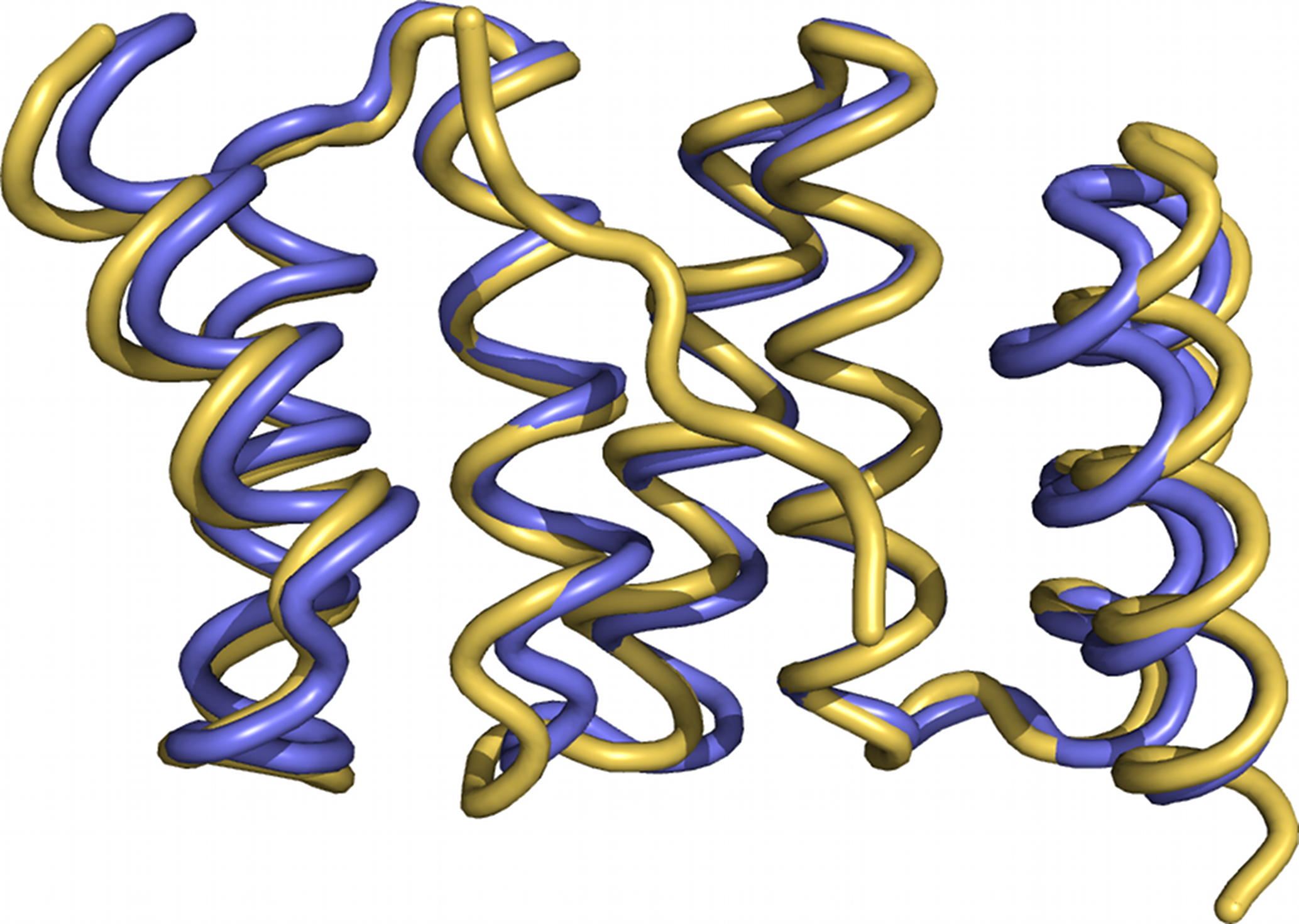

Supplement: Figure S2 — Overlay of the ITASSER modelled structure on the Hop crystal structure. Tube depiction of the superposition of the TPR model obtained from ITASSER on the high-resolution crystal structure of the TPR domain from Hop complexes with the Hsp70 octapeptide (1ELW). The modelled structure of the TPR domain is shown in blue color and that of the crystal structure is shown in yellow. The RMSD was found to be 1.36 Å, suggesting that the modeled structure was quite reliable for use in MD studies. (TIF) [file pone.0083461.s003.tif]

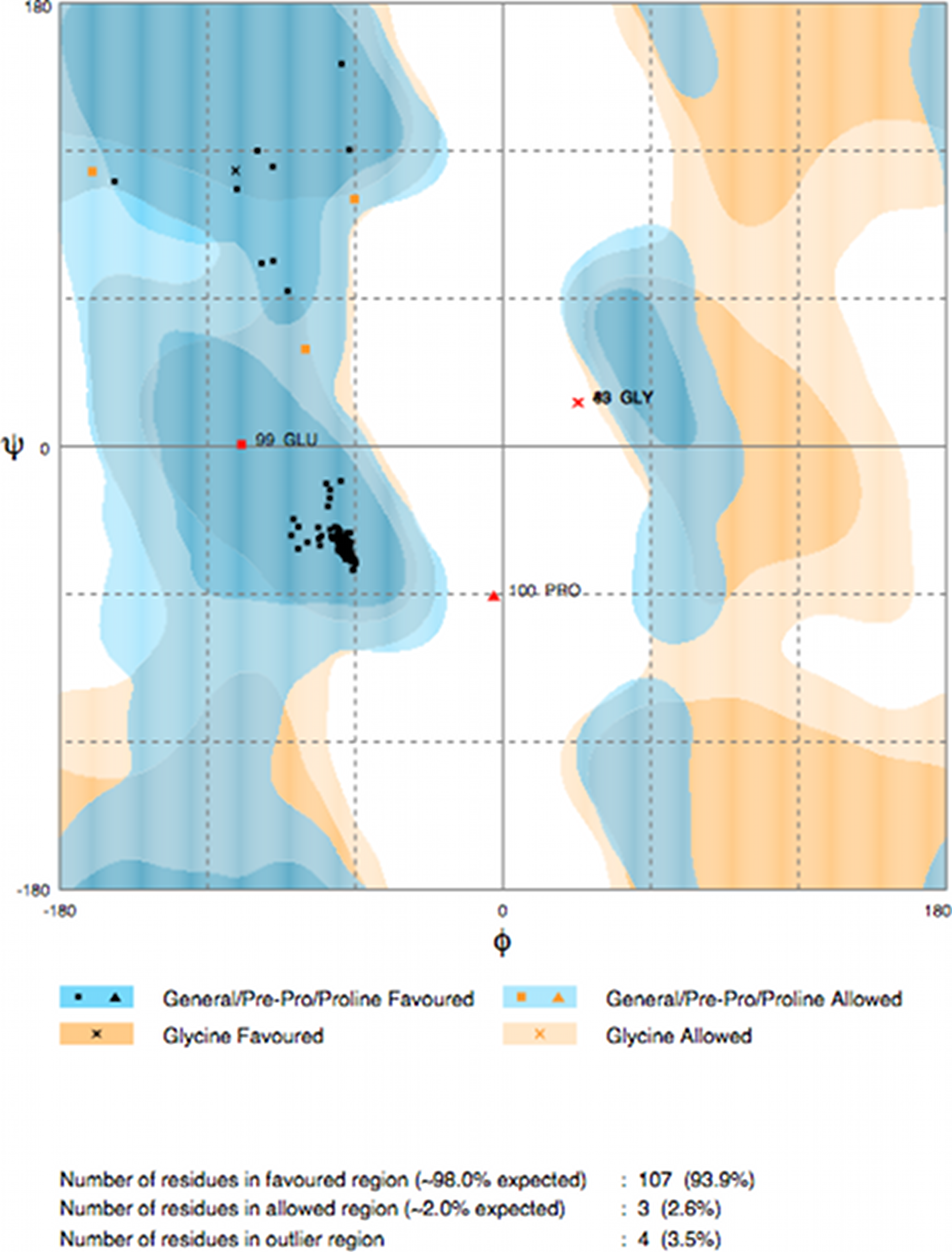

Supplement: Figure S3 — Ramachandran plot of modelled At Toc64_TPR (Apo). (TIF) [file pone.0083461.s004.tif]

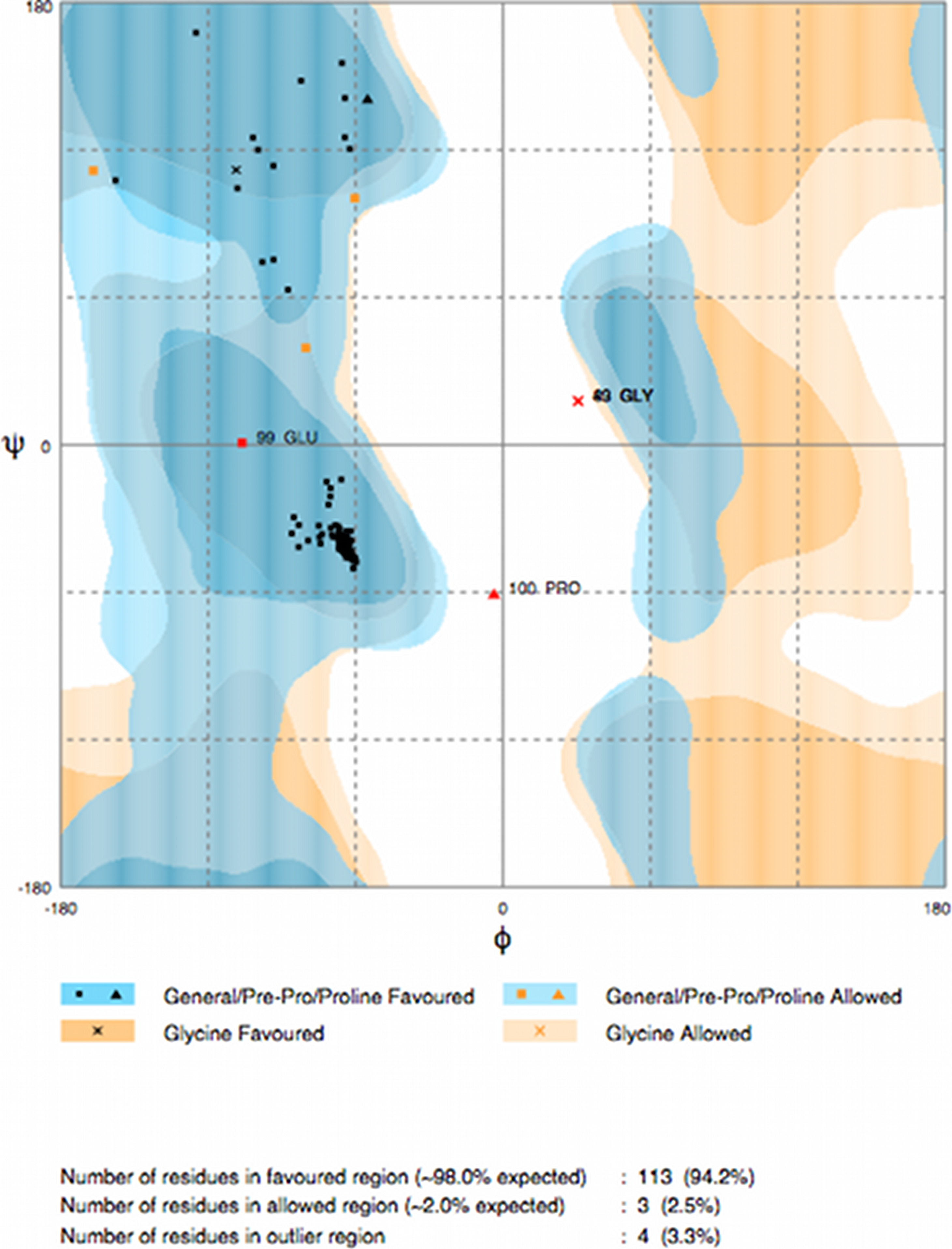

Supplement: Figure S4 — Ramachandran plot of C-Hsp70 (octapeptide) bound form (T_C70). (TIF) [file pone.0083461.s005.tif]

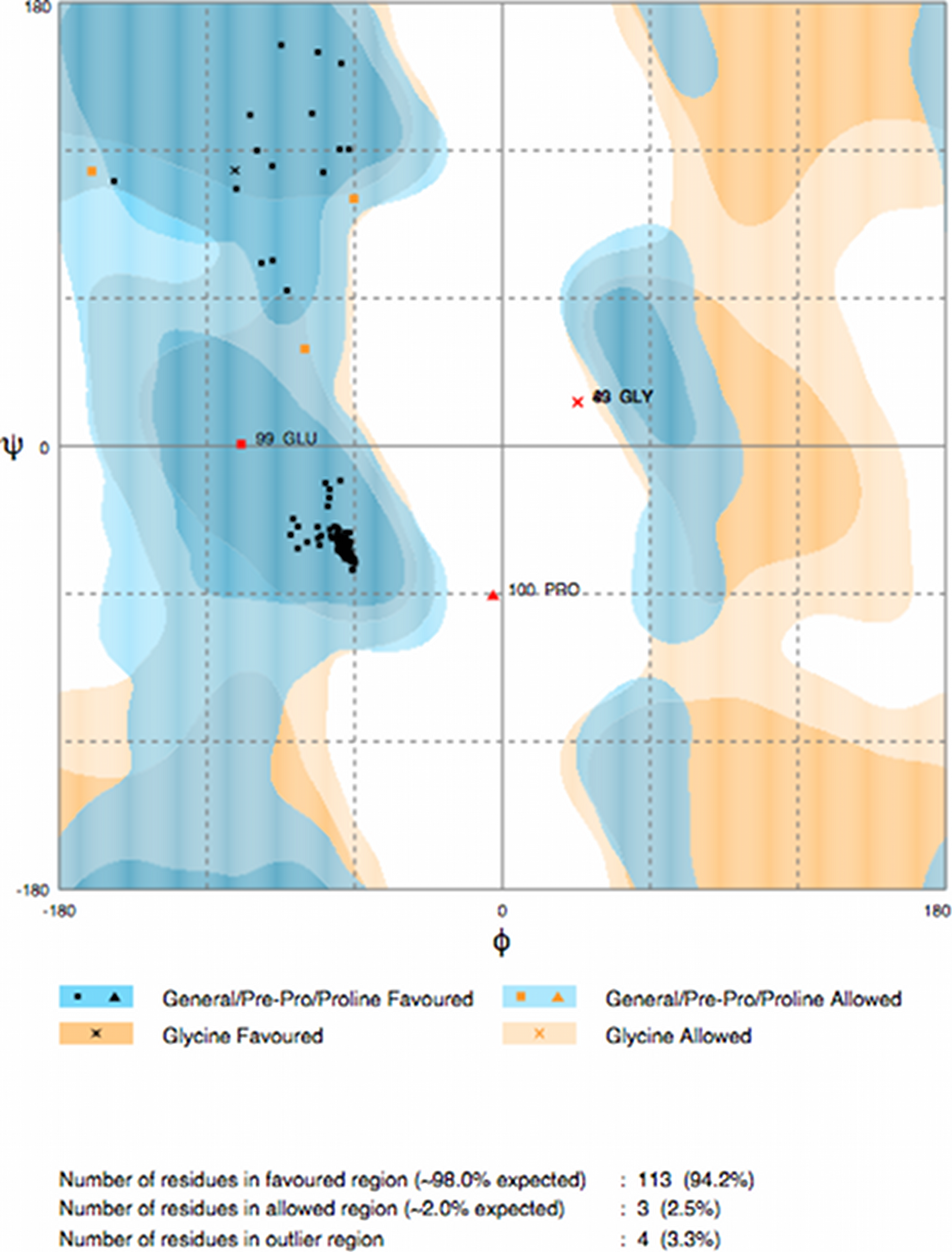

Supplement: Figure S5 — Ramachandran plot of C-Hsp90 (octapeptide) bound form (T_C90). (TIF) [file pone.0083461.s006.tif]

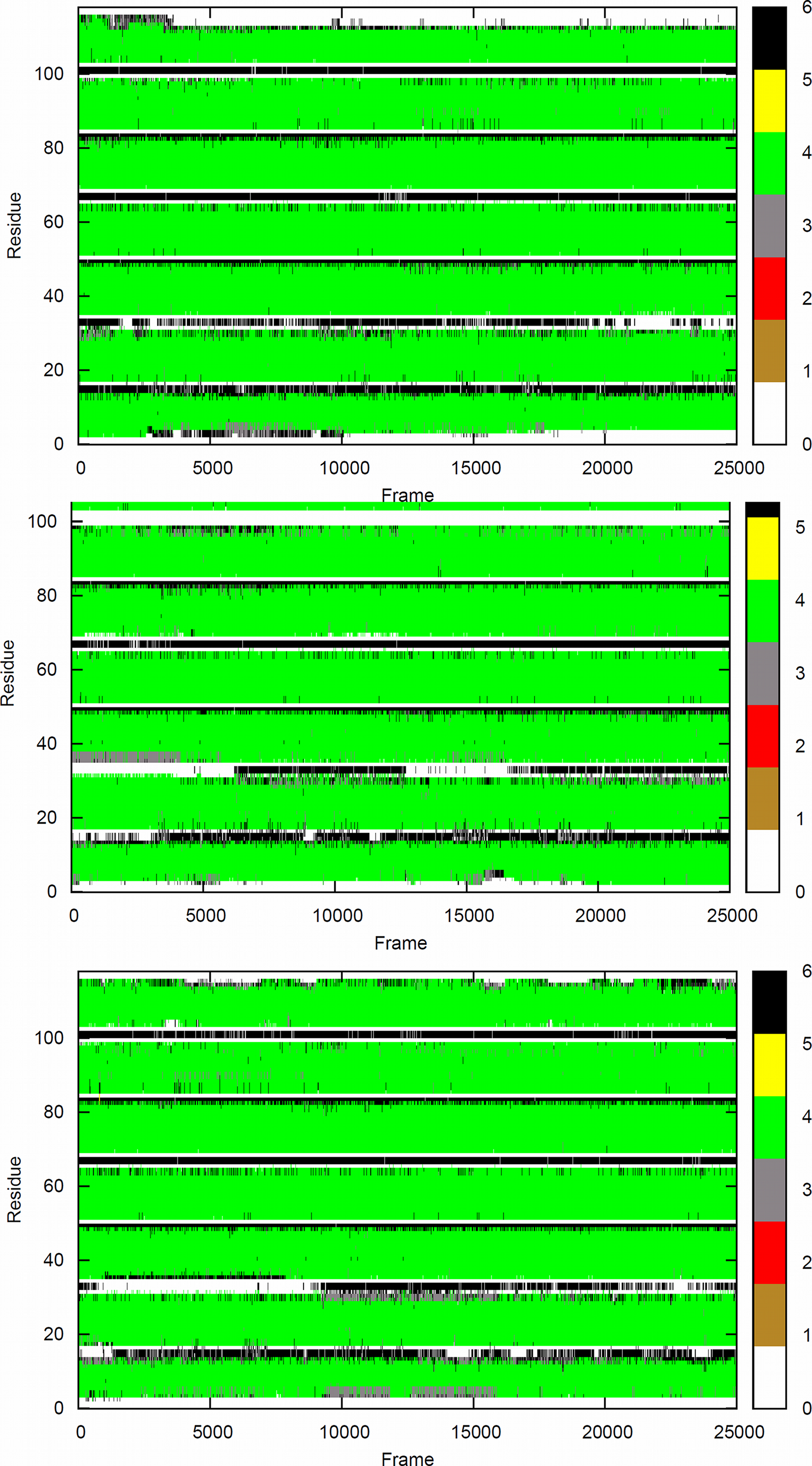

Supplement: Figure S6 — Secondary structure map of all the trajectories created by DSSP. Color codes: green represents alpha helices, black represents turns, grey represents 310 helices and white represents coils. (TIF) [file pone.0083461.s007.tif]

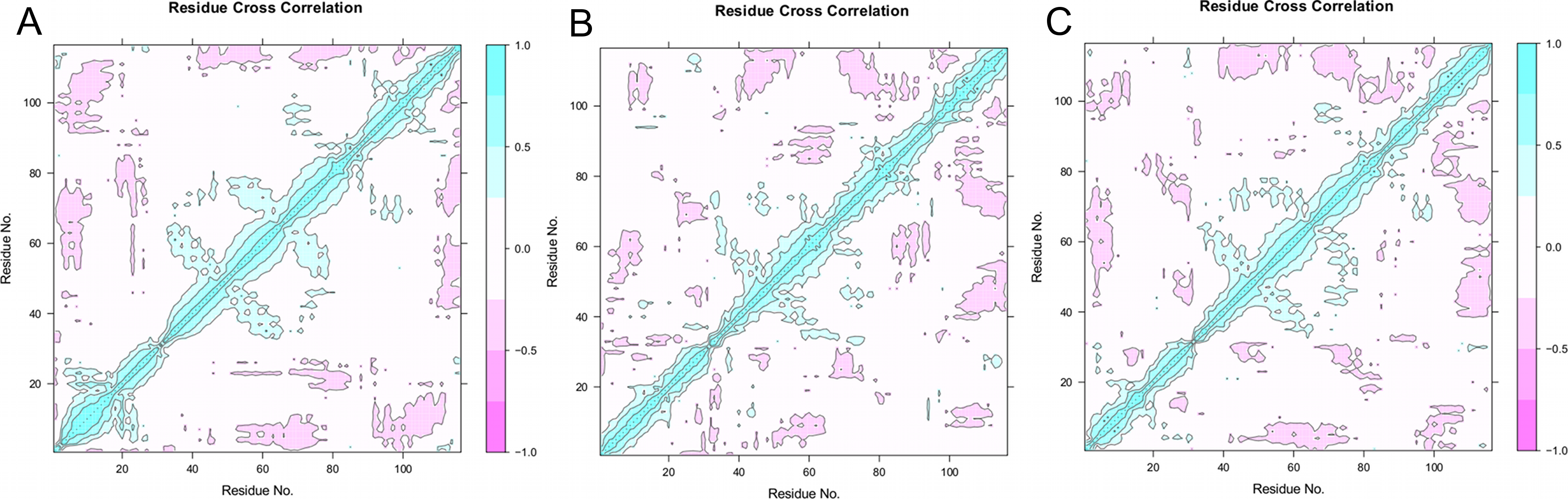

Supplement: Figure S7 — Dynamic cross-correlation maps. A. Apo, B. C-Hsp70 bound form of TPR and C. C-Hsp90 bound for of TPR using Cα atoms. (TIF) [file pone.0083461.s008.tif]

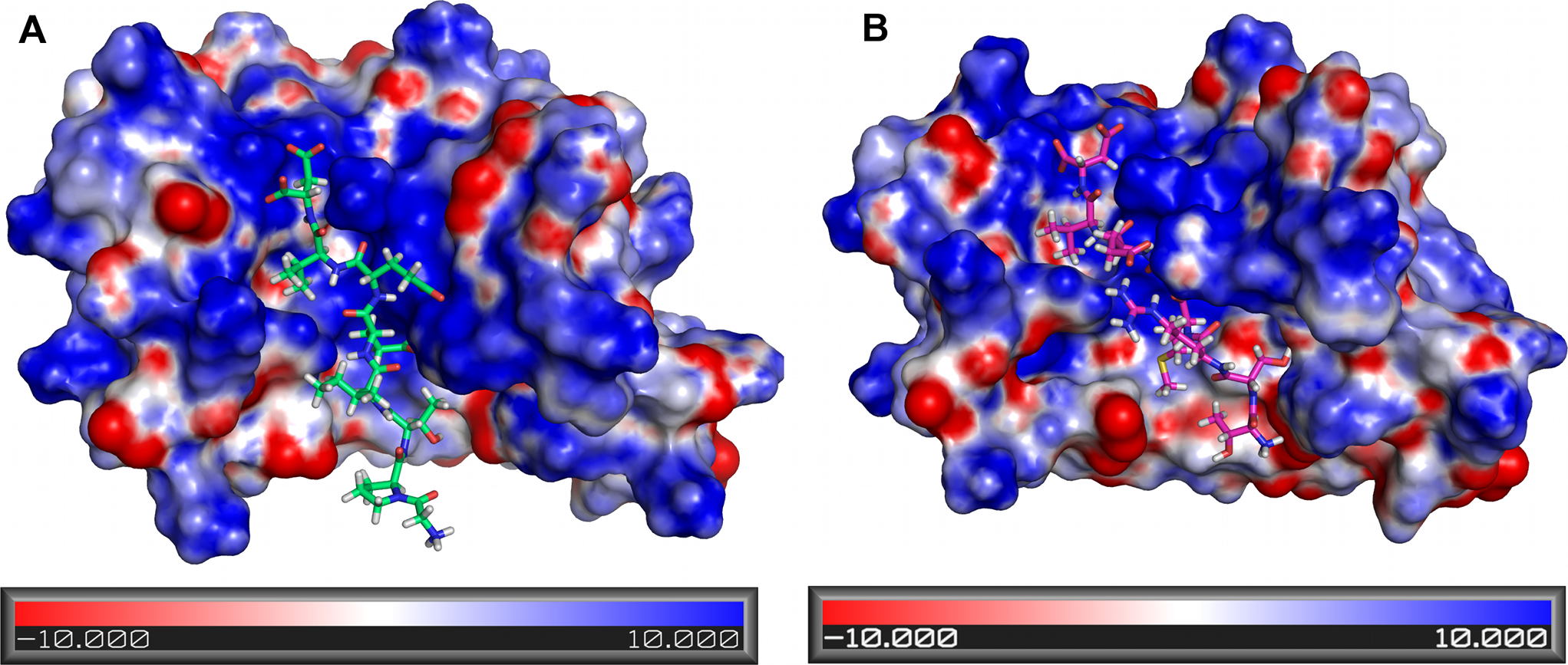

Supplement: Figure S8 — Molecular surface representations. A. C-Hsp70 octapeptide is bound to AtToc64_TPR; B. C-Hsp90 octapeptide is bound to AtToc64_TPR. The averaged pdb of the final 5ns of the simulation from each trajectory was used to create these maps. Electronegative and electropositive charges are colored in red and blue respectively. (TIF) [file pone.0083461.s009.tif]
